# Incentive Based Load Shedding Management in a Microgrid Using Combinatorial Auction with IoT Infrastructure

**DOI:** 10.3390/s21061935

**Published:** 2021-03-10

**Authors:** Bizzat Hussain Zaidi, Ihsan Ullah, Musharraf Alam, Bamidele Adebisi, Atif Azad, Ali Raza Ansari, Raheel Nawaz

**Affiliations:** 1 Department of Electrical Engineering, DHA Suffa University, Karachi 75500, Pakistan; bizzat.hussain@dsu.edu.pk; 2 Department of Electrical and Computer Engineering, CUI, Abbottabad Campus, Abbottabad 22060, Pakistan; ihsan@cuiatd.edu.pk; 3 Department of Electronics Engineering, Dawood University of Engineering and Technology, Karachi 74800, Pakistan; musharraf.alam@duet.edu.pk; 4 Department of Engineering, Manchester Metropolitan University, Manchester M15 5JH, UK; b.adebisi@mmu.ac.uk; 5 School of Computing and Digital Technology, Birmingham City University, Birmingham B4 7AP, UK; atif.azad@bcu.ac.uk; 6 Department of Mathematics and Natural Sciences, Gulf University of Science and Technology, Mishref Campus, Mubarak Al-Abdullah Al-Jaber Block 5, Masjid Al-Aqsa Road, Building 2 7207, Hawally 32093, Kuwait; Ansari.a@gust.edu.kw; 7 Department of Computing and Mathematics, Manchester Metropolitan University, Manchester M15 5JH, UK

**Keywords:** combinatorial auction, energy efficiency, evolutionary algorithms, load shedding, microgrid, smart grid, IoT

## Abstract

This paper presents a novel incentive-based load shedding management scheme within a microgrid environment equipped with the required IoT infrastructure. The proposed mechanism works on the principles of reverse combinatorial auction. We consider a region of multiple consumers who are willing to curtail their load in the peak hours in order to gain some incentives later. Using the properties of combinatorial auctions, the participants can bid in packages or combinations in order to maximize their and overall social welfare of the system. The winner determination problem of the proposed combinatorial auction, determined using particle swarm optimization algorithm and hybrid genetic algorithm, is also presented in this paper. The performance evaluation and stability test of the proposed scheme are simulated using MATLAB and presented in this paper. The results indicate that combinatorial auctions are an excellent choice for load shedding management where a maximum of 50 users participate.

## 1. Introduction

With the ever increasing population and growing industrial sector in the developing countries, providing a reliable energy service can be very difficult. With a wide gap in the supply capacity and the demand on the grid, a lot of investment is needed to improve the gird’s capacity to fulfil the demand of its users [1]. To solve this issue, a lot of efforts are now being made to replace the macrogrids with microgrids [2]. However, the major source of electricity generation for microgrids has been the renewable energy system, which often, is unable to fulfil the grid’s electrical demand. In such a scenario, a microgrid can have two possible solutions, (a) it can trade electricity from the other microgrids or the main grid or (b) it can curtail the energy released to its users to match supply capacity [2,3]. In this paper, we address the issue of load (or energy) curtailment or load shedding in a microgrid setting.

Previously, some work has been done to manage load shedding. For a fair load shedding, smart meters can be used to learn the patterns of energy consumption in households [1]. Additionally, the demand response of the households for load shedding can be modelled using two major approaches: (a) incentive based and (b) price based. Incentive based models are considered better in performance [3]; in such models the users curtail their loads for a pre-determined tariff [3]. Incentive based models have been proposed previously. Lee et al. gave an idea of using load curtailment as virtual generation, where a demand response service provider bade with the generators while giving load reduction instead of actual generation as the product [4]. Chai et al. used the principle of giving incentives to the customers in order to shift their load from the peak prices and in turn maximize the profit of the retailer [4].

However, the aforementioned work leaves significant scope for improvement. For example, no platform exists that selects the customers if several of them are willing to curtail load at the same time. This can allow to maximize the payment of load curtailment paid to different users as well as the profit for the grid operator. Moreover, all the previous works offer fixed incentives to all the customers instead of offering the incentives on merit. Furthermore, most of the works did not consider that a user can behave dynamically at different time periods, that is, the user’s willingness to curtail the load or afford a different price can change from time to time. In this paper, we propose an auction mechanism whereby the users can bid for energy curtailment. Therefore, below, we describe the precedent of using auction mechanisms in different scenarios in the power sector.

Since the arrival of smart grid and the concept of smart cities, a lot has changed on the technology front [5,6,7,8]. A lot of work has been carried out for the architecture of IoT for smart grids to collect the information, thus enabling a lot of different fields [9]. In [10,11], a three-layer structure containing device layer, application layer and network layer is discussed. Device layer (or the perception layer ) utilizes several kinds of different sensor tags and readers or sensor equipment to collect information. In [12], four layers are proposed: application layer, device layer, cloud management layer, and network layer. The device layer is further divided into two sub-layers: the first one being the thing layer to sense environment, control home appliances, and collect data and the second one being the gateway layer which controls how to establish a connection to the elements of thing layer. These advancements have helped in collecting and processing data from smart and micro grids for applications of energy trading and load management.

IoT devices have been introduced in homes and buildings in recent years in order to collect data on the building and its surroundings. These IoT devices can be used to collect several types of information and can be deployed on the installations (air handling unit (AHU), lift, chiller, etc.) to extract data such as temperature and vibration [13,14]. For the microgrid infrastructure, many communication protocols can be adopted. However, by applying different private protocols will result in poor interoperability and higher development costs. An alternative is the Internet of Things (IoT), an infrastructure of interconnected devices and systems, together with information resources and intelligent services. By using IoT to interconnect the devices within the microgrid, the system will become more intelligent and efficient. Moreover, the microgrid and energy management systems of the customers would no longer be stand-alone entities but part of a ubiquitous network.

Recently, a lot of efforts have been made on energy trading and its various applications. Energy trading has previously been done successfully for energy storage sharing and load sharing [15,16]. Trading methods for energy can be classified into two different categories, that is, the auction based approach and the game theoretic approach [16]. Auction mechanism is seen as a corner stone of the energy trading applications [7]. In this study, we have used auction mechanism as a platform to select participants in the load curtailment activity in order for them to gain some incentives in return. The main purpose of an energy auction is to find the lowest cost relation between demand and supply, and increase the overall social welfare, that is, the percentage sum of consumers’ surplus and producers’ surplus [17]. Competition in an energy auction motivates the users to go for energy saving devices and techniques such as demand response. However, most of the deployed auctions ignore the fact the at times bidders want to bid in compound ways, that is, they want to submit and win multiple bids at a time, in order to maximize their revenues. This problem is known as the exposure problem. A problem is defined as an exposure problem, when the will of the user is to win multiple unit of an item or wants to win several different items but end up winning too few [18]. According to different economist, exposure problem should be avoided in order to increase the efficiency of the auction process and increase the total revenue [18].

A Combinatorial auctions (CA) are touted as the best possible solution for the exposure problem. Using the properties of combinatorial auctions, bidders can place bids on individual items as well as combination of items present in the auction in form of packages [19]. The feature of package bidding helps combinatorial auctions in solving the exposure problem [20,21,22].

Despite their extensive use to solve the exposure problem, the combinatorial auctions have only sparingly been used in the field of energy trading that also faces the exposure problem. Penna et al. introduced the combinatorial auctions in electricity market and used them for seasonal electricity tariff [23]. Zaidi et al. used combinatorial auctions for multiple microgrid trading [2]. These auctions have also been used for allocation of common energy storage sharing and shared facility control application [5,15,16,23]. In this research, a reverse combinatorial auction has been used. An auction is said to be a reverse auction when it has multiple sellers and one buyer only. In our case, we have multiple sellers (the users willing to curtail electricity) and a single buyer (microgird). Each seller sells its load reduction for a price incentive. Using reverse combinatorial auction, the users can express their willingness in complex combinations in order to maximize their profits.

The winner determination problem of these auctions is defined to be as NP-Hard [2]. Historically, combinatorial auctions have been successfully been solved using Evalutionary algorithms (EA). EA’s ability to simultaneously exploit a number of solutions in a search space makes it a promising solution for solving various dynamic problems.

In this study, we use a hybrid algorithm that combines a genetic algorithm (GA) with Binary Particle Swarm Optimization (BPSO) to solve the winner determination problems (WDP) for the proposed reverse combinatorial auction. The Darwin’s theory of evolution is the main inspiration for Genetic Algorithms [24], which in turn define a class of evolutionary algorithms [17,25,26]. These Genetic Algorithms use techniques inspired by evolutionary biology such as mutation, inheritance, crossover and selection. Using social behaviour model-closely related to the swarming theory- of insects, fishes and birds as the main inspiration, Kennedy et al. proposed Partical Swarm Optimization, more commonly known as PSO [27,28]. Authors of [28], while comparing GA and PSO, concluded that the computational cost of both the algorithms is mainly problem dependent [29] gave a comparison of GA and PSO for solving unconstrained and constrained non-linear problems. The authors concluded that PSO works better in former problem type whereas, GA outperform PSO when exposed to the later problem types, However, studies have showed that despite some strengths and shortcomings or of both of the algorithms, hybridization yields better results for many problems in comparison to the standalone GA or PSO [27,28,30]; hybridization of metaheuristics is indeed common across a variety of evolutionary algorithms [31].

Both these methods have been extensively used for solving combinatorial auctions’ WDP. A Genetic algorithm based determination problem (WDP) is introduced in [32]. The bidders are only allowed to generate bid and offers in the XOR bid format because the use of OR and AND bid formats entails extra complexities and increases computational time. The PSO method is used in [33] for solving the WDP, but this produced suboptimal results. WDP in CA is similar to 0-1 knapsack problem and can be optimized using the algorithms used for solving Multi-dimensional Knapsack Problem [34]. In the past, hybrid meta heuristics have heavily been used to solve knapsack problem, in order to achieve optimality at a quicker rate [33,34,35].

The key contributions of this paper are:

- Idea of setting up a separate market for load curtailment within a microgrid environment with suitable IoT infrastructure.
- The idea of giving different incentives to various different users according to their bids for energy curtailment rather than fixed incentives for all.
- An auction mechanism for users to compete for load curtailment in a microgrid based on combinatorial auctions
- A winner determination solution for single sided reverse combinatorial auction for energy trading applications (one buyer multiple sellers).

This work is an extension of [2] where Zaidi et al. introduced combinatorial auction based multiple microgrid trading mechanism to enable trading in between microgrids, having IoT infrastructure. However, this paper focuses on how microgrids can manage their electricity need during peak hours, if they are not able to buy any electricity from other microgrids.

The paper organization is as follows: System model is presented in Section 2; Section 3 presents the overall auction process along with the winner determination process; a detailed simulation study is explained in Section 4; and finally, Section 5 concludes the paper.

## 2. System Model

Consider a microgrid consisting of n number of consumers such that i=1,2,3…,n. Each consumer is supposedly equipped with a load forecast system and energy storage system. Moreover, each day is divided into m time intervals such that j=1,2,3…,m. Each consumer $C_{i}$ expects to consume $Co_{ij}$ amount of energy at time interval *j*; however, at the same time it expects to reserve $D_{ij}$ amount of energy for energy curtailment, that is, it should be ready to curtail $D_{ij}$ energy whenever the grid needs. A microgrid manager (MGM) is connected with the consumers and is also equipped with the load and generation forecast system. When for time interval *j*, MGM predicts the shortfall of energy, it requests the auctioneer to start the auction for load curtailment. The auctioneer then sends the auction start notification to the consumers. MGC has a maximum reservation price $P_{j}$. Similarly, each consumer also has a minimum desirable incentive they are expecting for load curtailment.

**Figure 1:**
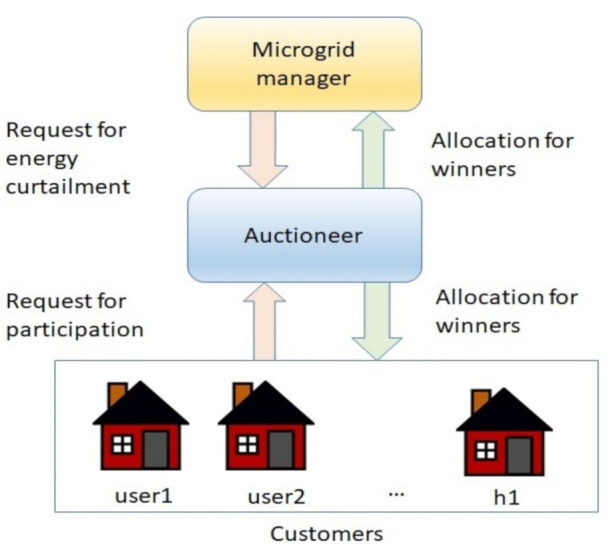
The system model for proposed mechanism.

Figure 1 shows the overall system model. The overall system is divided into three entities—buyer, sellers and auctioneer. In the proposed mechanism, MGM is the buyer, consumers are the sellers, whereas the auctioneer is a third party, who is responsible for gathering the bids from different entities, processing the winning bids and calculating the price of each trade. Provided with the load profile of the overall system and at the end based on combinatorial auctions a load curtailment market is set up.

In this study, the energy management system, the auctioneer, and the microgrid manager are all located within the same microgrid and are connected by either a wireless or a wired network. For the wireless networks, lightweight IP stacks and the IPv6-based communication protocol can be used to enable communication between the energy management systems of the customers and the auctioneer. For this purpose, 6LoWPAN [36,37] can be applied to low-power devices with limited processing capabilities allowing them to participate in the IoT infrastructure.

## 3. Overall Auction Process

### 3.1. Main Entities

**Figure 2:**
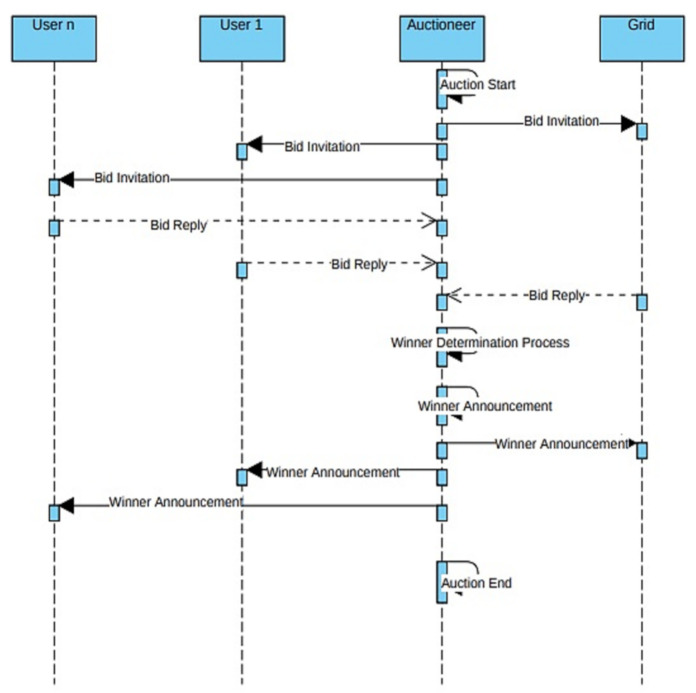
Overall auction process.

The buyer, sellers and the auctioneer are the three main entities involved the auction process. Users act as sellers as they are selling their capability to sell load curtailment, grid becomes the buyer, which buys users ability to curtail load at a certain given incentive. Whereas, auctioneer is the central figure which controls this trade between users and the grid. Figure 2 shows the overall auction process. At the start of the auction, the auctioneer collects the bids from the grid and users and arranges them in accordance to their order design. After bids initialization, winners are selected using the WDP. The WDP of the combinatorial auctions improves the overall social welfare. The buyer, sellers and the auctioneer are the three main entities involved the auction process. At the start of the auction, the auctioneer collects the bids from the buyer and sellers and arranges them in accordance to their order design. After bid initialization, winners are selected using the WDP. The WDP of the combinatorial auctions improves the overall social welfare.

### 3.2. Structure of the Auctioneer

**Figure 3:**
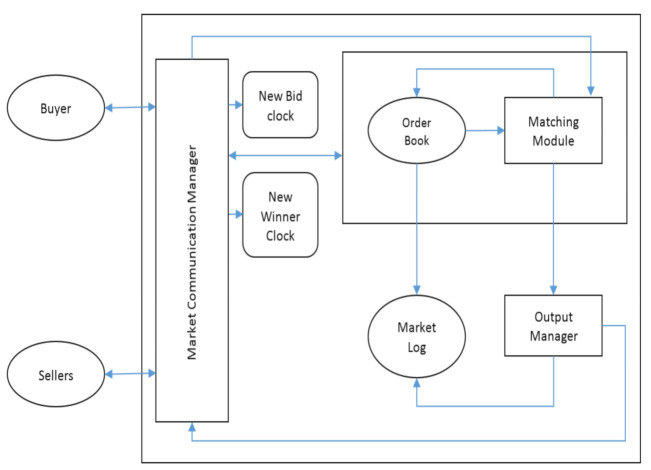
Structure of the auctioneer.

Figure 3 shows the overall structure of the auctioneer. The key components of auctioneer are as follows:

- Market Communication Manager: For Communication between auctioneer and bidders. It collects the bids, informs the bidders about the results, communicates with the matching module via order book and output manager.
- New Bid Clock: Keeps an eye on new bids. If the timer runs out, the winners are announced and round of auction is concluded. Refreshes to the initial stage, whenever there is a new bid is received
- New Winner Clock: Keeps a tab on new Winner. Refreshes whenever there is a new winner (buyer and sellers selected for trade).
- Matching Module: Runs the Winner Determination algorithm and selects the winners. Looks for new winners until round of auction has ended. Gets the bids from market logs and announce the results through output manager and communication manager.
- Order Book: All the bids are collected in the order book and remains there until they win or are expired.
- Market Output Manager: Gets the results from the matching module and store them in market log, while also giving the results to users via market communication manager
- Market Log: Keeps the history of the market trades, all the winning and non-winning bids which (valid and expired bids) via order book and output manager. Provides historical data to the users and the grid.

### 3.3. Social Welfare

The percentage sum of consumers’ surplus and producers’ surplus is said be as the social welfare [5]. This can be expressed as

(1) $$\;S.W.=(\Sigma Con_{sur}+\Sigma Pro_{sur})\;$$

(2) $$\;Con_{sur}=\frac{\left(Con_{will}-Will_{acual}\right)}{Con_{will}}\;$$

(3) $$\;Pro_{sur}=\frac{\left(Will_{actual}-Pro_{will}\right)}{Pro_{will}}\;$$

where, S.W. is the social welfare, $Con_{sur}$ is consumer’s surplus, $Pro_{sur}$ is the producer’s surplus, $Con_{will}$; in EUR; is the price the consumer is willing to pay, $Pro_{will}$; in EUR;is the minimum price the producer is willing to get, and $Will_{actual}$ in EUR, is the trading price determined by the auctioneer.

### 3.4. Bid Configurations for Submission

The idea of package bidding or combinations is used in the combinatorial auctions. In package bidding, bidders are entitled to submit more than one bid, according to their needs and optimal function, at any time [20,21,22]. In this study, users are permitted to bid in order configuration using OR bids, XOR bids and atomic bids [20,21,22].

### 3.5. Winner Determination Process

Integer programming can be used to express the winner determination problem also termed here as utility or fitness function. In this study, solving the winner determination problem means maximizing the overall load reduction for the MGM along as well as the increasing the incentives for the consumers. The winner determination is given by,

(4) $$max\sum_{j=1}^{m}\sum_{i=1}^{n}H_{i,j}$$

such that,

(5) $$\sum_{j=1}^{m}\sum_{i=1}^{n}H_{i,j}\leq Sh$$

(6) $$\sum_{j=1}^{m}P_{j}\geq \sum_{j=1}^{m}\sum_{i=1}^{n}TP_{i,j}$$

(7) $$H_{i}=D_{i}.a_{i}$$

(8) $$TP_{i,j}\geq RP_{i,j}$$

where, $H_{i,j}$, measured in KW represents all the accepted load reductions, $D_{i}$ is the load reduction bade by the individual customer *I*, whereas $a_{i}$ shows whether the bid is accepted or rejected; $a_{i}$ can be either 1 or 0. If any part of the bid is accepted, the value of $a_{i}$ becomes 1. $P_{j}$, in EUR is the grid (buyer)’s maximum reservation price for all hours of the day, whereas, $TP_{i,j}$, in EUR, is the incentive price allotted to customer *i* at time *j*. Sh is the maximum value of curtailment in KW, needed at time *j*. Furthermore, $RP_{i,j}$, in EUR is the minimum reservation price of the users (sellers).

The winner determination problem (WDP) of combinatorial auctions is considered to be NP-Hard problem. Furthermore, it is similar to the 0–1 knapsack problem (KP) [38,39]. A KP problem occurs when resource allocation must obey different constraints. Initially, single-unit winner determination problem was equated to weighted set packing problem [34]. However, authors of [39] discussed a relationship between winner determination and knapsack problems [39]. Since, KPs—which are intensively studied in the past—are relatively easy to understand; solving CA as 0–1 KP has been a common practice, To solve the 0–1 KPs, the use of meta-heuristics has been frequent [40]. As combinatorial problems require larger search space as compared to other optimization problems; thus, Meta-heuristics such as EAs have been seen as an ideal solution.. Additionally, EA’s ability to simultaneously exploit a number of solutions in a search space makes it a promising solution for solving various dynamic problems. It is well known that the research on WDP algorithms has profited from the algorithms used for Multi-Dimensional KP [34,39]. Many of previous studies have considered EA for winner determination of combinatorial auctions by mapping the WDP as KP [29,32]. However, these works have their limitations as well; for example, the time taken to find the optimal solution is large, or the optimal solution is not found at all. This is because in combinatorial auctions, each and every combination possible is checked similar to the KP; this produces a large search space. Hybrid Meta-heuristics are able to find the optimal solution for such problems and effectively solve the WDP of combinatorial auctions [2,32]. In this study, we use a hybrid algorithm that combines a genetic algorithm (GA) with Binary Particle Swarm Optimization (BPSO) to solve the winner determination problems for the proposed reverse combinatorial auction. In the past, hybrid meta heuristics have heavily been used to solve knapsack problem, in order to achieve optimality at a quicker rate [33,34,35].

### 3.6. Proposed Algorithms

**Figure 4:**
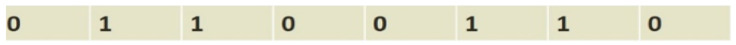
Population Type.

**Figure 5:**
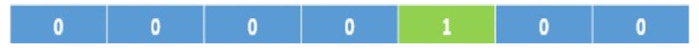
Bid selection structure in Binary form.

The BPSO algorithm is an iterative method that we leverage to optimise the objective function defined in Equation (4) subject to Equations (5)–(7). BPSO starts by randomly generating a swarm of particles, where each particle is a binary string as shown in Figure 4 and Figure 5. A signle particle is depicted in Figure 4. Particles indicate a suggested solution in terms of accepted and rejected bids, as shown in Figure 5, where bid acceptance is represented using 1 and 0 shows a rejected bid. While generating the swarm, OR and XOR bids have the most priority, while the atomic bids have the least priority. In BPSO, each particle has a position vector and a velocity vector. A particle’s position encodes a candidate solution to the problem at hand (a combination of bids in our case). Since the current position (or the solution) is not necessarily optimal, the PSO iteratively changes the position of each particle such that the average quality of the solutions in the swarm improves. The velocity of each particle represents the magnitude and the direction of change in its position in the next iteration.

Initially, as stated earlier, each particle k is assigned a random position $z_{k}$ and a random velocity $v_{k}$ [2]. Each particle’s fitness value (that is the quality of the solution that it represents) is calculated using the objective function defined in Equation (4) subject to Equations (5)–(8); as the fitness values are evaluated for each particle, the personal best position found for each particle and the global best position for the entire swarm are updated. The personal best position indicates the best fitness value of each particle (so far given the changes in its position), whereas the global best position indicates the best fitness value amongst all the particles. This process is run until the saturation point is reached. A saturation point is defined as the stage after which there is no change in the global or personal best positions. The particles with the lower fitness values are discarded and the global best solution along with other fit particles are taken to the next step.

After the above process, a group of particles with the highest fitness values, that is, the elite particle group, is further refined via a Genetic Algorithm (GA). Following the GA terminology, each particle’s position vector is now called a chromosome. Instead of using velocity vectors to manipulate these chromosomes, these chromosomes are now manipulated via the artificial genetic operators (Appendix B), that is, mutation and crossover to form new chromosomes [5]. As earlier in the PSO, the fitness of each chromosome resulting from the artificial genetic operators is calculated using the winner determination objective defined in Equation (4) subject to Equations (5)–(8). The process is repeated until the saturation point is reached. The final successful bid matches are represented by the chromosome with the best fitness value. The overall winner determination process is shown by Algorithm 1.
**Algorithm 1:** Hybrid GA and BPSO For Combinatorial Auction (AUCGENPSO)# Binary Particle Swarm Optimization **Initialize wpopulation**     Priority Order        OR-Bids        XOR- Bids        Atomic Bids **Repeat**     Calculate particle’s position and velocity      Calculate Fitness Function using Equation (4) **Until saturation is reached** # Genetic Algorithm     Select the set of fittest particles from PSO to initialise the GA population **Repeat**     Perform reproduction using crossover and mutation     Calculate fitness function using Equation (4)     **Until saturation is reached** **END**


## 4. Experimental Study

### 4.1. Simulation Scenario

We assume that 50 households are connected in a microgrid environment. The users are connected to the microgrid controller via an auctioneer. The microgrid is equipped with a generation capacity of 350 KW to 400 KW. The households bid for the load reduction for incentives at different times of the day using combinatorial auction mechanism. Profile of households along with the price data was taken from [41,42]. MATLAB R2015a was used to implement the proposed auction mechanism. Using a Matlab based bid generator, combinatorial bids were generated for the simulation purpose [43]. The simulation results are compared to the sequential double auction [44]. The load profiles, showing average and maximum load of the participating users are shown in Table 1.

### 4.2. Simulation Analysis

The simulations analyses conducted in this study are described next.

#### 4.2.1. Average Load Profile

**Figure 6:**
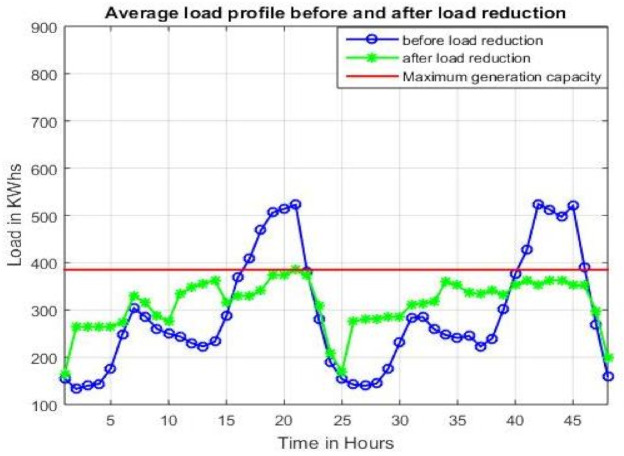
Average load profile before and after load reduction is presented.

The overall load profile of the micro-grid is compared in two cases; (a) without any load reduction and (b) with load reduction. As illustrated in Figure 6, the red line shows the maximum generation capacity of the microgrid, so it is necessary for the load to stay below this line. However, the original load profile peaks at around 20 and 42 h, which in turn requires load shedding. However, after the load reduction, these peaks stay under the maximum generation capacity and the load is successfully shifted at other times, that is, between 10 and 15 h and 25 and 40 h. Moreover, in the load profile after load reduction, the overall load stays below the maximum generation time for the entire time, thus eliminating the load-shedding scenario at all times.

#### 4.2.2. Load Reduction

**Figure 7:**
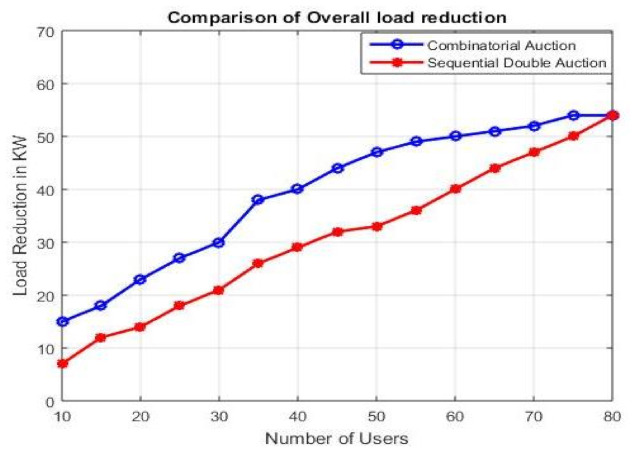
Overall load reduction.

In this subsection, we compared the load reduction done using combinatorial auctions and Sequential Double Auction. As illustrated in Figure 7 , the amount of load reduction increases as the number of users increases. Furthermore, the load reduction with respect to the number of users is better for combinatorial auctions than for sequential auctions (Appendix A) for up to 65 users. However, the load reduction through combinatorial auctions got saturated around 75 users, highlighting its limitation for larger number of users. Whereas, load reduction through sequential auctions maintained an increasing trend.

#### 4.2.3. Average Incentives

**Figure 8:**
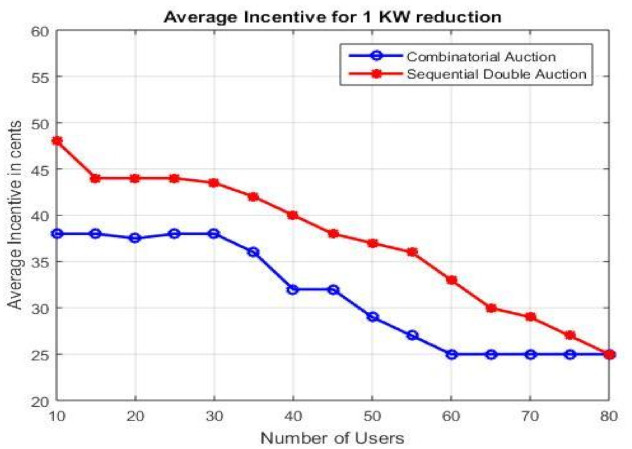
Average incentive per KW reduction.

In this subsection, we compared the average incentives per unit for combinatorial and sequential double auctions. As shown in Figure 8, as the number of users are increased, the amount of incentive per KWh is decreased because of the increase in number of winners. Moreover, the results of combinatorial auctions are better than the sequential auctions. Though, the combinatorial auctions saturate around 55 user but have a better results than sequential auction for up to 75 users. This concludes that for a region where number of users are less than 75, combinatorial auctions are good choice and vice versa.

#### 4.2.4. Social Welfare

**Figure 9:**
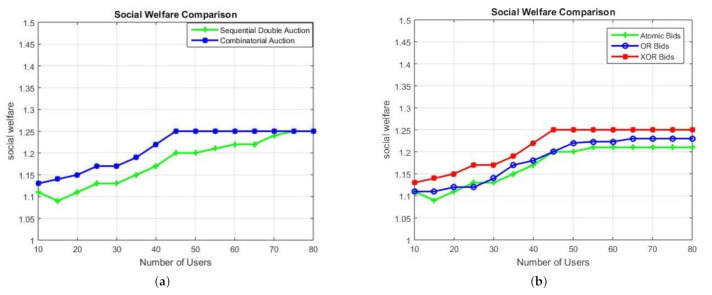
(**a**) Social welfare comparison (**b**) social welfare comparison for different bid types in combinatorial auctions.

In this subsection, we have compared the social welfare, explained in Equations (1)–(3), of the auction. In Figure 9, the social welfare of combinatorial auctions is compared to the social welfare of the sequential double auction. For up to 65 users, at 1.25(25%) the overall social welfare is better for the combinatorial auctions than at 1.2(20%) for the sequential double auction case. Figure 9 shows the comparison of social welfare for different bid types used in the combinatorial auctions, that is, OR, XOR and the atomic bids. The XOR bids have the highest social welfare value of 1.27 whereas, OR has the maximum figure of around 1.22. The atomic bids have a maximum social welfare value of up to 1.2. Moreover, it was also noted that the overall social welfare value saturates around 45 to 50 number of users for the combinatorial auction. Thus, making the proposed combinatorial auctions a good choice for up to 50 users.

#### 4.2.5. Optimality Analysis of Proposed Algorithm

**Figure 10:**
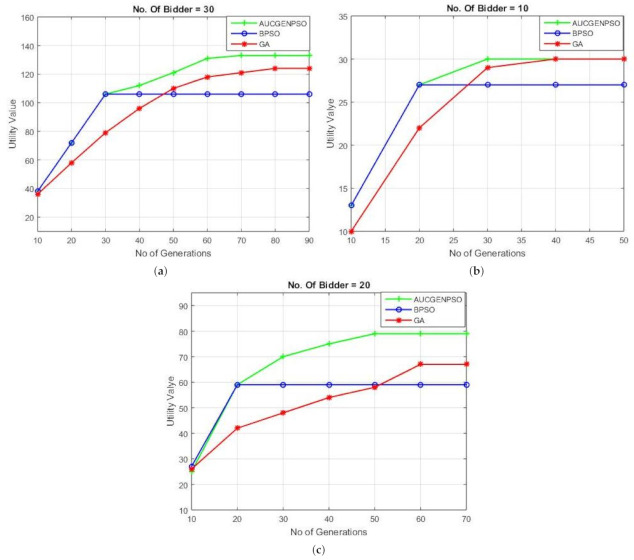
Optimality results (**a**) no. of bidders = 10, (**b**) no. of bidders = 20, (**c**) number of bidders = 30.

Under this heading, we compare our the proposed technique, with BPSO and GA, with the number of generations and the fitness value or generally iterations as the main parameter for performance evaluation. An optimal solution, having low number of iterations and high fitness value, was the desired outcome. While increasing the number of users from 10 to 30, the number of generations required to obtain the optimal fitness value were observed. A comparison between the proposed algorithm with GA and BPSO is depicted in Figure 10. BPSO, while converging early for up to 30 users , has a lower fitness value as compared to GA, which takes the highest time to converge However, AUCGENPSO clearly outperforms GA as it converges early with a higher fitness value.

## 5. Conclusions

Load shedding is a major issue especially with the ever growing power demand. With the advancement in IoT technology, it is easier for the grids to ask users to curtail load at the time of peak demand. In this paper, we focused on a scenario where in a microgrid environment, the service provider hands out incentives to customers to curtail their load during the peak hours. Problem from the service provider’s end was discussed in this paper and was solved using auction mechanism to select the customers to participate in load curtailment in order to gain some incentives. For better efficiency of the system, combinatorial auctions were used. The proposed method was shown to be useful in a microgrid environment by performing the performance evaluation of the overall auction process. Moreover, it was noted that the social welfare improved as the number of participants increased.

This work can be further extended by adding non-financial incentives along with the financial incentives. Furthermore, many different combinations such as AND can also be included in the future studies. Finally, other machine learning algorithms [45] can also be explored, compared and contrasted.

## Figures and Tables

**Table 1:** Load profiles of households.

Household	1	2	3	4	5	6	7	8	9	10	11	12	13	14	15
Avg. Load (KW)	2.32	4.26	4.3	3.13	4.76	3.9	4.06	4.17	3.8	3.79	4.03	4.45	5.71	5.54	4.49
Max Load (KW)	3.72	4.57	5.81	4.61	6.25	5.73	5.35	5.2	5.29	4.94	5.182	5.76	6.82	6.21	5.42
Household	16	17	18	19	20	21	22	23	24	25	26	27	28	29	30
Avg. Load	2.83	3.78	4.9	4.8	3.9	3.84	5.6	4.03	5.44	4.23	4.38	3.771	3.81	4.74	5.44
Max Load	3.96	4.51	5.67	5.72	4.94	4.81	6.3	4.97	6.31	5.09	5.21	4.67	4.89	5.64	6.23

**Figure A1:**
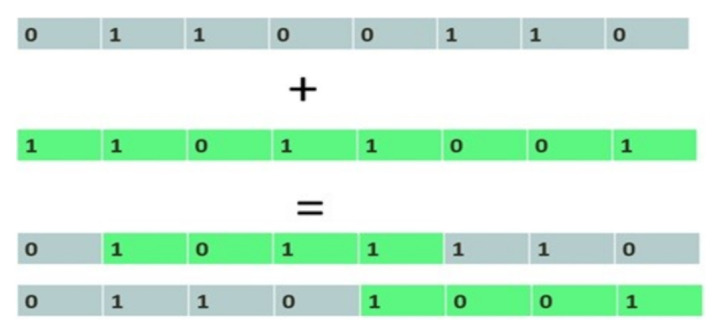
CrossOver Mechanism.

**Figure A2:**
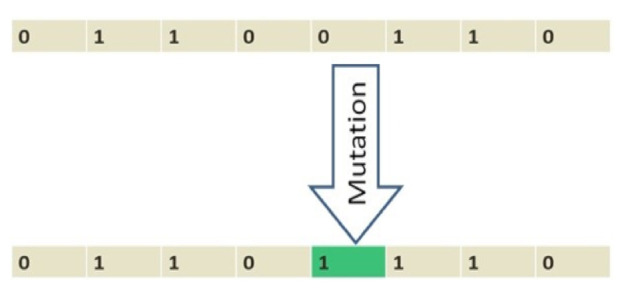
Mutation Mechanism.

## Data Availability

Not applicable.

## Appendix A. Sequential Auctions

A scenario where multiple players share a common ES plant was established. Sequential auctions is organized at different time intervals t. Supplier submitted its supply bids including the minimum price at which it is willing to trade and each player submitted their demand bid, which includes the maximum price at which they can trade. Then, by using supply and demand curves, the uniform clearing price and trading volume are identified. Buyers who bid more than the maximum clearing price are allowed to buy storage rights [41].

## Appendix B. Genetic Operators

### Appendix B.1. Crossover Mechanism

Crossover is a process to combine two or more solutions (or chromosomes as they are called in the GA parlance) to create new solutions [24]. In this study, we have used the uniform crossover mechanism. In the uniform crossover mechanism, corresponding genes (bit positions) of two parent chromosomes are exchanged to form two new (or offspring) chromosomes. Figure A1 exemplifies the crossover mechanism used in this study. In this example, the offspring particles have half the information from each of the parent particles arranged in a random manner.

### Appendix B.2. Mutation Mechanism

In mutation, one or more chromosome gene values are altered randomly [24]. Figure A2 shows the mutation mechanism used in this study. One chromosome representing bid matches is changed to a new one using a bit-flip mutation (0 is flipped to 1). This forms a new set of chromosomes and hence increases the variability of the population.
